# Beehive-Derived Products for Wound Management with a Focus on Diabetic Wounds: Mechanistic Insights, Advanced Delivery Strategies, and Translational Evidence

**DOI:** 10.3390/ph19101632

**Published:** 2026-10-14

**Authors:** Raj Kamal, Abhishek Chauhan, Hiti Narula, Ankit Awasthi, Mohit Kumar, Sonia Dhiman, Neeraj Choudhary, Dinesh Kumar, Emad M. Abdallah

**Affiliations:** 1 Chitkara College of Pharmacy, Chitkara University, Rajpura 140401, Punjab, India; raj.kamal@chitkara.edu.in (R.K.); abhishek.chauhan@chitkara.edu.in (A.C.); hiti@chitkara.edu.in (H.N.); ankit.awasthi@chitkara.edu.in (A.A.); mohit.5167@chitkara.edu.in (M.K.); sonia.dhiman@chitkara.edu.in (S.D.); 2 GNA School of Pharmacy, GNA University, Phagwara 144401, Punjab, India; dineshpotlia123@gmail.com; 3 Department of Biology, College of Science, Qassim University, Buraydah 51452, Saudi Arabia

**Keywords:** beehive products, bee venom, apitherapy, antimicrobial activity, antioxidant activity, anti-inflammatory effects, nano-formulations, wound dressings

## Abstract

Wound healing is a complex biological process that can be severely impaired by persistent inflammation, oxidative stress, microbial infection, impaired angiogenesis, and defective extracellular matrix remodelling. These abnormalities are particularly prominent in diabetic wounds but also contribute to delayed healing in burn, venous, pressure, traumatic, and post-surgical wounds. Beehive-derived products, including honey, propolis, royal jelly, beeswax, and bee venom, have attracted considerable interest as multifunctional natural therapeutics because of their diverse antimicrobial, antioxidant, anti-inflammatory, immunomodulatory, and regenerative activities. Honey supports wound repair through its acidic pH, high osmolarity, hydrogen peroxide generation, and bioactive phytochemicals, whereas propolis provides flavonoids and phenolic compounds that contribute to antimicrobial, antioxidant, and tissue-regenerative effects. Royal jelly promotes cellular proliferation, angiogenesis, and tissue repair, while beeswax primarily provides moisture retention, barrier protection, and structural properties in wound dressings. Bee venom and its major component, melittin, exhibit antimicrobial, anti-inflammatory, and regenerative effects, although dose-dependent toxicity and hypersensitivity remain important concerns. Recent advances in hydrogels, polymeric films, electrospun nanofibres, nanoparticles, and composite scaffolds have improved the stability, local retention, controlled release, and therapeutic performance of these products. Preclinical studies demonstrate enhanced antimicrobial activity, fibroblast migration, collagen deposition, angiogenesis, and wound closure, while emerging clinical evidence supports their potential across several wound types. However, variability in composition, standardisation, safety, formulation stability, and limited large-scale clinical evidence remain major challenges.

## 1. Introduction

The natural healing of a wound is a tightly regulated biological process that includes the sequential steps of haemostasis, inflammation, proliferation and tissue remodelling [1]. However, various acute and chronic wounds, including diabetic foot ulcers (DFUs), venous leg ulcers, pressure ulcers, burn wounds, traumatic injuries, and post-surgical wounds, frequently exhibit impaired healing due to persistent inflammation, excessive oxidative stress, microbial colonisation, biofilm formation, impaired angiogenesis, and defective extracellular matrix (ECM) remodelling [2]. Diabetic wounds are one of the most difficult clinical problems, as hyperglycaemia has an aggravating effect on such pathological changes, leading to prolonged hospitalisation, re-infection, high cost of treatment and a significant risk of lower-limb amputation [3]. Although wound care has improved, current therapies fail to take into account the multiple factors involved in impaired wound healing and highlight the need for multifunctional therapeutic strategies [4]. Natural products have gained considerable attention as alternative and complementary therapies because of their broad spectrum of biological activities and favourable safety profiles [5]. Beeswax, honey, propolis, royal jelly, bee venom and others are among these, which are promising candidates for wound management. Due to their natural composition, which is rich in phenolics, flavonoids, enzymes, peptides, fatty acids, vitamins and minerals, they have strong antimicrobial, antioxidant, anti-inflammatory, immunomodulatory and regenerative activities [6]. Beehive products act on several pathological processes related to impaired wound healing at the same time, in contrast to conventional single-target therapies, and this makes them especially attractive to treat both acute and chronic wounds [7].

Honey is the most widely studied product of the beehive used as a wound treatment and is very effective in the following actions. It maintains a moist wound environment, promoting autolytic debridement, lowering the wound microbial burden due to its acidic pH, high osmolarity, hydrogen peroxide production and phytochemical composition, and stimulating granulation tissue formation [8]. Made up of a combination of bee-collected resin from plant exudates, propolis contains high amounts of flavonoids and phenolic acids, which have strong anti-inflammatory, antimicrobial and antioxidant properties, along with the ability to stimulate the proliferation of fibroblasts, collagen synthesis, and tissue remodelling [9]. Royal jelly is rich in bioactive peptides, 10-hydroxy-2-decenoic acid (10-HDA) and major royal jelly proteins (MRJPs), which are known to stimulate cell proliferation, angiogenesis and immune regulation [10]. Beeswax provides structural strength and moisture barrier properties in wound dressings [11], while bee venom has shown antimicrobial, anti-inflammatory, and regenerative effects in specific doses [12].

In recent years, with the development of biomaterials and pharmaceutical formulations, the therapeutic potential of these natural products has been further extended [13]. The incorporation into hydrogels, electrospun nanofibres, polymeric films, nanoparticles, nanoemulsions, and composite scaffolds has enhanced their stability, controlled release, bioavailability and localised delivery to the wound site. The advanced delivery systems not only prevent degradation of bioactive constituents but also allow for sustained delivery and maintenance of an optimal wound microenvironment, thereby improving the therapeutic efficacy [14]. The incorporation of apitherapeutics into current wound management materials is a potential opportunity to create multifunctional wound management products that are able to prevent infection, suppress inflammation and oxidative stress, promote angiogenesis and tissue repair [15]. This review summarises all the pathophysiological mechanisms involved in impaired healing of wounds and critically evaluates the therapeutic effects of the major products produced by beehives, such as honey, propolis, royal jelly, beeswax and bee venom. In addition, it reviews recent developments in the delivery systems of nanoengineered and biomaterials, summarises the current preclinical and clinical data for various types of wounds and indicates ongoing challenges and future prospects for the successful translation of apitherapeutic formulations to effective clinical interventions for wound management.

### Scope, Novelty and Distinction from Previous Reviews

Recent reviews have highlighted the therapeutic potential of bee-derived products in wound care, while others have specifically focused on honey-containing hydrogels and advanced natural-bioactive wound dressings. However, the present review provides a broader and more integrated perspective by comparatively examining the major beehive-derived products, including honey, propolis, royal jelly, beeswax, and bee venom, within a unified wound-healing framework. Particular emphasis is placed on their molecular and cellular mechanisms involving inflammatory regulation, oxidative-stress control, antimicrobial and antibiofilm activity, angiogenesis, re-epithelialization, and extracellular matrix remodelling. Importantly, the available evidence is stratified according to wound model and translational relevance, with specific attention to diabetic and chronic wound conditions while clearly distinguishing supportive evidence derived from acute and non-diabetic wound models. In addition, this review integrates conventional biological evidence with emerging delivery approaches, including hydrogels, nanofibres, films, nanoparticles, and other advanced biomaterial-based systems designed to improve local retention, controlled release, stability, and therapeutic performance of beehive-derived bioactives. Recent developments in redox regulation, antimicrobial and biofilm control, responsive wound-healing hydrogels, controlled polyphenol delivery, and regenerative tissue-engineering strategies are also considered to provide a contemporary translational perspective. Collectively, this integrated evaluation of multiple beehive-derived products, mechanistic pathways, evidence hierarchy, advanced delivery systems, and wound-specific translational relevance distinguishes the present review from previous literature focusing predominantly on individual bee products or particular wound-dressing technologies.

## 2. Pathophysiology of Diabetic Wounds

**Figure 1:**
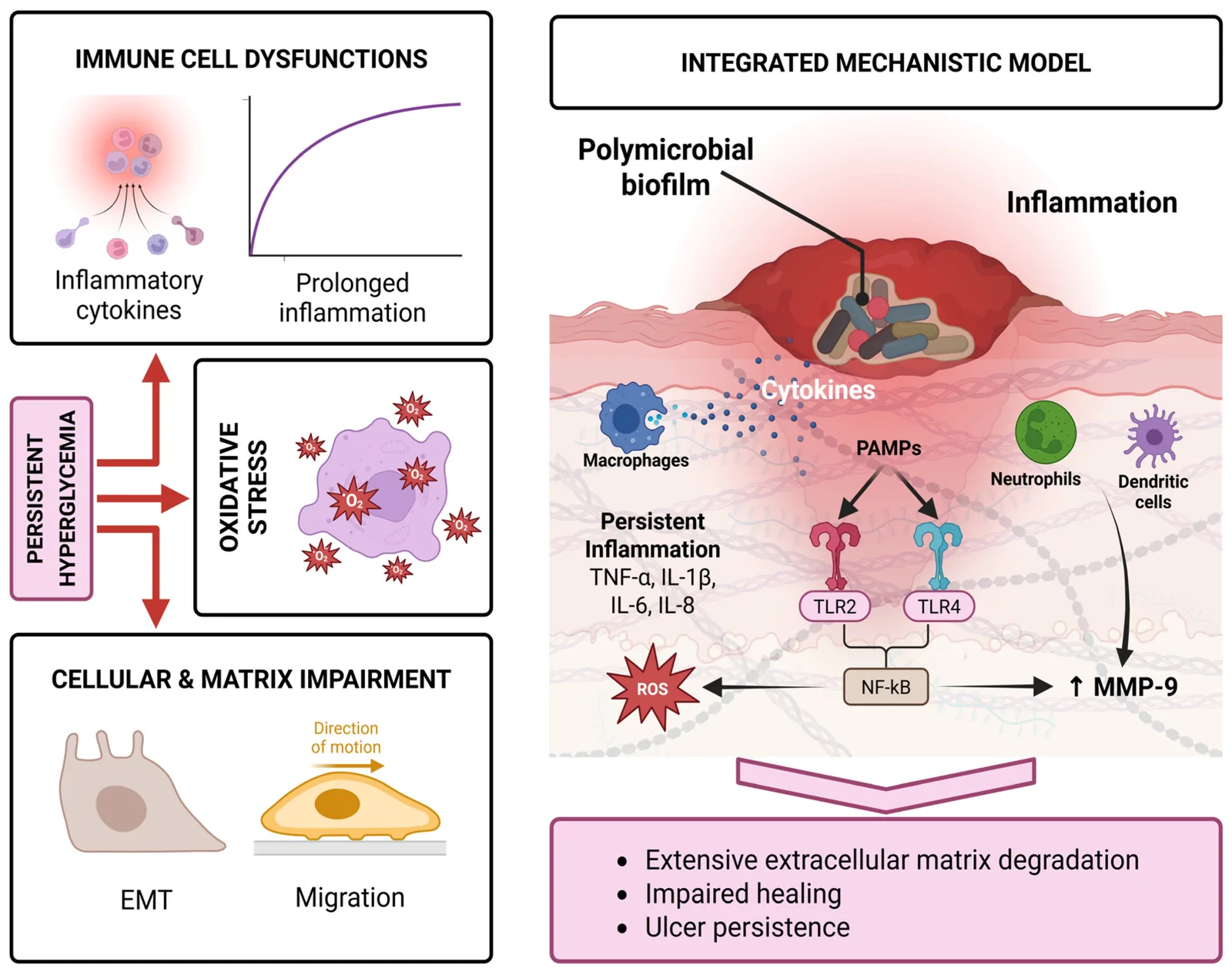
Integrated mechanisms underlying impaired wound healing, highlighting the roles of persistent hyperglycaemia, immune dysfunction, oxidative stress, microbial biofilm formation, inflammation, and cellular and extracellular matrix impairment. Created in BioRender. Ishaya, J. (2026) https://BioRender.com/c1n7a6s.

Impaired wound healing is a multifactorial condition associated with metabolic, vascular, immunological, microbial and ECM abnormalities, all of which contribute to deranged inflammatory, proliferative and remodelling stages of wound healing [16], as illustrated in Figure 1. The mechanisms are different for each type of wound. However, there are certain common features of the pathological changes, such as continued inflammation, oxidative stress, microbial infection, inadequate angiogenesis, and reduced ECM remodelling [17]. Diabetic hyperglycaemia also contributes to these abnormalities, as it causes delayed re-epithelialisation of diabetic wounds and inhibits the function of keratinocytes, fibroblasts, endothelial cells and immune cells, while promoting deposition of advanced glycation end products (AGEs) [18]. This continuous activation of neutrophils and macrophages causes chronic wounds to enter a prolonged inflammatory state where a surplus of pro-inflammatory cytokines, matrix-degrading proteases, and reactive oxygen species (ROS) are produced [19]. Chronic oxidative stress disrupts the function and viability of fibroblasts, collagen production, and leads to a hostile environment that impairs tissue regeneration [20]. At the same time, impaired immune function, tissue hypoxia, vascular insufficiency and high glucose levels make the host more vulnerable to microbial colonisation by microbes that can easily develop biofilms, which shield the bacteria from host immune function and antimicrobial therapy [21]. In addition, endothelial dysfunction and impaired vascular endothelial growth factor (VEGF) signalling impede angiogenesis and tissue oxygen supply to the wound [22]. Additionally, excessive action of matrix metalloproteinases (MMPs) and degradation of ECM compromise collagen remodelling and tissue integrity. Chronic inflammation, oxidative stress, microbial biofilm formation, impaired angiogenesis and defecti-ve ECM remodelling together will not allow for effective tissue regeneration and will lead to a chronic non-healing wound that will remain highly susceptible to infection and severe complications [23].

### Evidence Stratification According to Wound Model and Translational Relevance

The therapeutic evidence for beehive-derived products should be interpreted according to the experimental or clinical wound model [24]. This is because DFUs represent a complex chronic wound environment characterised by hyperglycaemia, oxidative stress, impaired angiogenesis, prolonged inflammation, and increased susceptibility to microbial colonisation and biofilm formation [25]. Direct evidence includes diabetic animal models, hyperglycaemia-associated cellular models, and clinical studies involving diabetes-related wounds, providing greater relevance to DFUs [26]. In contrast, studies using healthy-animal, burn, surgical, oral, nipple, tendon, and other non-diabetic wound models should be considered supportive or extrapolated evidence because they do not directly establish efficacy in DFUs [27]. Therefore, findings from acute healthy-animal models should not be considered equivalent to efficacy in chronic diabetic ulcers. Greater emphasis should be placed on outcomes relevant to diabetic wounds, including control of inflammation, oxidative stress, microbial burden and biofilms, restoration of angiogenesis, ECM remodelling, and cellular migration. Clinical evidence should likewise be interpreted according to wound type and study design, while registered trials without available results should not be considered evidence of therapeutic efficacy [28]. The strength of evidence also depends on study design and methodological quality. In-vitro and animal studies provide mechanistic and preclinical evidence, whereas case reports, pilot studies, and observational studies provide preliminary clinical evidence. Randomised controlled trials provide comparatively stronger clinical evidence; however, their reliability remains dependent on adequate sample size, randomisation, blinding, appropriate controls, and risk-of-bias assessment (Table 1).

## 3. Overview of Beehive Products and Their Bioactive Constituents

**Figure 2:**
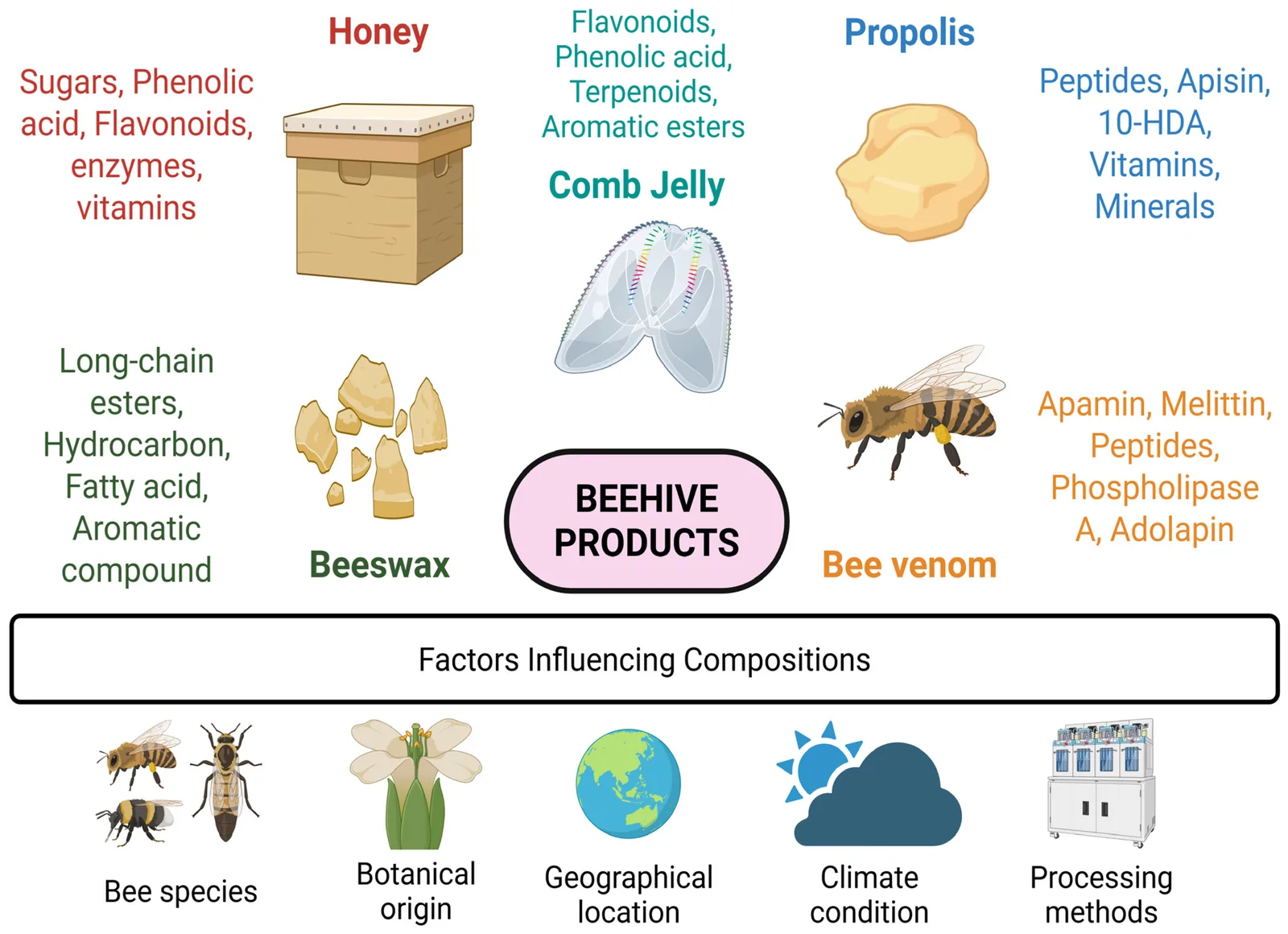
Overview of major beehive products, their principal bioactive constituents, and factors influencing their composition, including bee species, botanical origin, geographical location, climate, and processing methods. Created in BioRender. Ishaya, J. (2026) https://BioRender.com/c1n7a6s.

Beehive products are all-natural compounds made by honeybees and have long been used in traditional medicine (Figure 2) but have recently come to be known for their therapeutic effects in wound care [29]. Such products contain a wide range of bioactive components such as carbohydrates, proteins, lipids, phenolic compounds, flavonoids, enzymes, peptides, vitamins, minerals, as well as volatile phytochemicals, all of which are responsible for various antimicrobial, antioxidant, anti-inflammatory, immunomodulatory, and regenerative effects [30]. The chemical composition varies depending on the bee species, plant origin, geographical location, climatic conditions, and processing methods, but each beehive product has a distinct phytochemical profile that is responsible for its biological activity. Beehive products have proved to be effective natural therapeutics and important ingredients of advanced wound dressings and biomaterial-based delivery systems, as they target several pathological mechanisms associated with diabetic wound healing simultaneously [31].

### 3.1. Honey

Honey is the most extensively explored product from the beehive that is used for wound healing and is composed primarily of sugars (fructose and glucose) and water [32]. In addition to many bioactive compounds, all of these are responsible for the therapeutic properties of the honey [33]. They are phenolic acids (caffeic, p-coumaric, ferulic and ellagic acids), flavonoids (chrysin, pinocembrin, quercetin, apigenin and galangin), enzymes (glucose oxidase and catalase), organic acids, vitamins, minerals, amino acids, peptides and volatile compounds [34]. When diluted, glucose oxidase catalyses the slow production of hydrogen peroxide, which results in sustained antimicrobial activity while not causing any significant tissue toxicity. Furthermore, honey has a low pH, high osmolarity and is rich in phenolics, which explains the wide range of antibacterial, antioxidant, anti-inflammatory and immunomodulatory properties. These properties help to keep the wound moist, promote autolytic debridement, stimulate granulation tissue and promote re-epithelialization [35]. The antimicrobial activity of honey is mediated by multiple complementary mechanisms. High osmolarity limits water availability for microbial growth, while glucose oxidase generates hydrogen peroxide (H_2_O_2_), contributing to antimicrobial activity [36]. Its acidic pH further restricts microbial growth, whereas methylglyoxal (MGO), particularly in Manuka honey, provides an additional antibacterial mechanism. Honey-derived defensin-1 also contributes to antimicrobial defence [37]. In parallel, phenolic compounds and flavonoids can reduce oxidative stress and modulate inflammatory responses, thereby supporting the wound-healing microenvironment.

Recent investigations have also confirmed the therapeutic effectiveness of honey for diabetic wounds. McArdle et al. (2023) investigated medical-grade honey (MGH) at three different pH levels (6, 7 and 8) against some clinically relevant microorganisms such as Staphylococcus aureus (SA), Staphylococcus epidermidis (SE), Escherichia coli (EC) and Pseudomonas aeruginosa (PA). The study showed that wound pH does not significantly affect the antimicrobial activity of medical-grade honey against wound micro-organisms, confirming the ability of MGH to maintain its antibacterial activity at all pH levels, thus proving that MGH can be safely used in both diabetic foot ulcers and post-surgical wounds [38]. Similarly, Agbagwa et al. [39] investigated the antibacterial efficacy of eleven Nigerian honey samples in 54 diabetic Wistar rats randomly allocated into nine experimental groups (*n* = 6/group). The highest antibacterial activity of the tested samples was observed in moringa-infused honey, which gave a diameter of the inhibition zone of 21.5 ± 2.12 mm compared with 15.0 ± 2.12 mm for other formulations and 13.0–13.5 mm for several other honey samples against Staphylococcus aureus. On day 3, day 6 and day 9, the wounds contracted significantly in the groups treated with honey, and catalase activity increased significantly in most of the groups treated with honey, which is a good indicator of antioxidant defence. Moringa-enriched honey was found to have a synergistic effect with honey’s antimicrobial activity and antioxidant properties compared with 1% silver sulfadiazine in the diabetic wound healing process [39]. The antimicrobial action of honey has also been expanded to cover wound infections associated with biofilms. Skadiņš et al. [40] tested 40 monofloral Latvian honeys against *E. coli*, *P. aeruginosa*, *S. aureus*, MRSA and *C. albicans*. Of these, 11 samples of honey had antibacterial activity against all the bacterial species tested, and they were more effective against the Gram-positive bacteria. In addition, the selected samples also inhibited the development of biofilm and exhibited activity against preformed biofilm, which indicated that polyphenol-rich honey has the potential to be an excellent natural antimicrobial biomaterial in the treatment of chronic wounds [40].

Studies have also been carried out on the effect of botanical source on the bioactivity of honey. Muñoz et al. [41] compared three Ulmo honey samples containing 52.77%, 68.41%, and 82.80% Eucryphia cordifolia pollen. Significant differences were observed in physicochemical characteristics, including pH, moisture, total sugars, and total solids. Histological examination revealed that all wounds treated with the different samples reached the proliferative phase, and the re-epithelialization process was completed or partially completed after 10 days, showing that the nutrient composition of Ulmo honey did not significantly affect the wound healing process [41]. Chrysostomou et al. [42] conducted a systematic review and meta-analysis to compare the efficacy of MGH to conventional wound-care products, such as silver dressings, povidone-iodine, enzymatic agents, absorbent dressings and larval therapy. The authors concluded that MGH dressings represent one of the most versatile wound-care options because of their combined antimicrobial and pro-healing properties, safety, ease of application, and cost-effectiveness, recommending their use in both acute and chronic wounds, particularly in elderly patients with difficult-to-heal ulcers [42].

### 3.2. Propolis

Propolis is a resinous substance gathered by honeybees from plant buds and exudates and combined with beeswax and enzymes produced by the bees [43]. It is rich in flavonoids, phenolic acids, aromatic esters, terpenoids, steroids and other polyphenolic compounds, which account for its various pharmacological properties. Some of its main bioactive components, such as caffeic acid phenethyl ester (CAPE), chrysin, galangin, pinocembrin, quercetin and artepillin C, are recognised as important mediators of antioxidant, antimicrobial, anti-inflammatory and cytoprotective activity [44]. Propolis also has anti-oxidative properties, inhibits pro-inflammatory signalling pathways, has antimicrobial properties, stimulates proliferation of fibroblasts, enhances collagen deposition and promotes ECM remodelling. It is one of the most promising natural compounds for diabetic wound therapy [45]. In recent years, the multifunctional therapeutic properties of propolis in wound care have been further demonstrated. Necip et al. (2024) extracted several phenolic compounds from eco-friendly white bee propolis and then tested the biological activities using gingival fibroblast cells, finding them to be fraxetin, apigenin, galangin, pinobanksin and chrysin. The propolis water extract clearly showed the ability to enhance the oral mucosal wound healing process and demonstrated strong antioxidant activity relative to untreated controls. Furthermore, the isolated phenolic compounds showed significant biological activity by inhibiting acetylcholinesterase (IC_50_: 0.45 ± 0.01 to 1.15 ± 0.03 mM), indicating additional pharmacological activity; however, this finding should not be considered direct evidence of wound regeneration [46]. Similarly, Balderas-Cordero et al. (2023) investigated the wound-healing efficacy of a 10% (*w*/*v*) Chihuahua ethanolic extract of Mexican propolis (ChEEP) using a full-thickness mouse wound model. The topical formulation was not toxic in the short term and had antibacterial properties against Staphylococcus epidermidis and Staphylococcus aureus. Additionally, ChEEP showed a marked anti-inflammatory effect in a TPA-induced mouse ear oedema model after 14 days of treatment. It also enhanced wound contraction, wound healing time, wound tensile strength and increased the deposition of type I collagen, indicating that the synergistic effect of polyphenols was effective in accelerating tissue repair [47]. The effect of geographical origin was also studied on propolis bioactivity. Dekebo et al. (2024) compared three 70% ethanolic propolis extracts from the Andong field, Gongju field, and Gongju mountain regions of Korea. The major flavonoids identified by GC-MS were pinocembrin (12.0–17.7%), chrysin (5.2–6.8%), and apigenin (5.30–5.84%). Among the tested preparations, the 10% Gongju mountain propolis ointment showed superior wound-healing activity, while differences in antioxidant activity were observed among the propolis preparations. The wound-healing property of the 10% Gongju mountain propolis ointment was superior to the other groups in excision, incision, and dead-space wound models. Furthermore, the anti-inflammatory properties of propolis, by inhibiting the production of inflammatory mediators (iNOS, IL-1β, IL-6) and wound-healing properties, which were determined by measuring cell migration, were strongest for Gongju mountain propolis, revealing that the wound-healing, antioxidant and anti-inflammatory activity of propolis is strongly affected by its regional variation in terms of its flavonoid composition [48].

In another study, Conceição et al. (2022) assessed two topical formulations with 1% Brazilian red propolis for the Wistar rat excision wound model. Histological and immunohistochemical evaluation demonstrated better wound contraction, faster wound epithelialisation, decreased crust formation, and increased wound healing biomarkers such as collagen I, collagen III, transforming growth factor-β3 (TGF-β3), and vascular endothelial growth factor (VEGF). In addition, propolis caused a significant rise in endogenous antioxidant enzymes (SOD, glutathione (GSH), glutathione reductase (GR), and myeloperoxidase (MPO)) with responses similar to those obtained in collagenase-treated controls [49]. More recently, Hernandez-Hernandez et al. (2025) compared hexane, chloroform, and methanolic extracts of Apis mellifera propolis from Michoacán, Mexico. The methanolic extract showed the highest antibacterial activity, with Staphylococcus aureus being the most sensitive microorganism, and also showed strong antifungal activity against Candida albicans and Trichophyton mentagrophytes. The wound-healing effect of the methanolic extract was found to be higher than that of the commercial positive control (Recoveron^®^), showing antioxidant activity of 12.23 μg/mL, total phenolic content of 580 mg GAE/g and total flavonoid content of 12.35 mg QE/g. Chemical analysis also identified flavanols, flavones and flavanones, demonstrating the role of phenolic compounds in the antimicrobial, antioxidant and regenerative properties of propolis [50].

In diabetic wound models, topical propolis has demonstrated effects beyond general antioxidant and antimicrobial activity. In a pilot study involving 24 patients with diabetic foot ulcers, topical propolis reduced ulcer area by 41% at week 1 compared with 16% in matched controls (*p* < 0.001), while active MMP-9 in wound fluid decreased by 18.1% versus 2.8% at week 3 (*p* < 0.001). Bacterial counts were also significantly reduced. These findings suggest that propolis may influence the inflammatory and proteolytic environment characteristic of diabetic wounds [51]. Hyperglycemia promotes excessive ROS generation and oxidative tissue injury, which can impair diabetic wound repair. Propolis has demonstrated antioxidant activity in diabetic models; for example, water-soluble propolis derivatives reduced lipid peroxidation and DNA damage in diabetic mice. In addition, topical propolis reduced IL-1β, IL-6, TNF-α and MMP-9 levels in diabetic wound models, suggesting potential modulation of oxidative and inflammatory pathways [52]. Altogether, these results show that propolis has a wound-healing potent effect by several complementary mechanisms, such as its potent antimicrobial and antioxidant activity, its ability to inhibit inflammatory mediators, its stimulating effect on the proliferation of fibroblasts and collagen synthesis, its stimulating effect on angiogenesis and its effect on the regulation of ECM remodelling. These diverse biological functions render propolis an interesting natural compound to be included in the design of advanced dressings and nano-enabled delivery systems in diabetic wound healing treatment.

### 3.3. Royal Jelly

Royal jelly is a nutrient-rich secretion of the worker bees used to feed the queen bee and the developing larvae [53]. It contains water, proteins, carbohydrates, lipids, vitamins, minerals, free amino acids and numerous bioactive peptides. The main functional components are major royal jelly proteins (MRJPs), 10-hydroxy-2-decenoic acid (10-HDA), apisin, royalisin, jelleines, defensin-1 and other antimicrobial peptides [54]. These bioactive molecules show potent antioxidant, antimicrobial, immunomodulatory and regenerative activities through stimulation of the proliferation of fibroblasts, migration of keratinocytes, angiogenesis, collagen synthesis and the remodelling of ECM [55]. Additionally, royal jelly regulates inflammatory signalling pathways, reduces oxidative stress and promotes upregulation of cellular metabolism, which helps repair chronic and diabetic wounds. Recently, Álvarez et al. (2023) showed that extracellular vesicles extracted from royal jelly (RJEVs) express exosomal markers (CD63 and syntenin) as well as proteins that are biologically active, such as MRJP1, defensin-1, and jellein-3. These vesicles changed the differentiation and secretory activity of the MSCs and inhibited lipopolysaccharide-induced inflammation in macrophages by inhibiting the MAPK signalling pathway. Furthermore, the extracellular vesicles of RJEVs showed antibacterial effects and wound healing activity was enhanced significantly in a splinted mouse wound model, indicating the role of extracellular vesicles in the wound healing activity of royal jelly [56].

Diabetic wound models have also been used to investigate the therapeutic potential of royal jelly. Lin et al. (2026) examined the effects of protein hydrolysates from Castanea mollissima royal jelly (CmRJPH) on advanced glycation end product (AGE)-damaged keratinocytes and lipopolysaccharide (LPS)-activated macrophages. CmRJPH strongly promoted the proliferation and migration of keratinocytes, improved the impaired migration of keratinocytes under diabetic conditions and reduced macrophage inflammation mediated by the inhibition of the NF-κB signalling pathway. As a result, the levels of inflammatory mediators such as iNOS, COX-2, IL-6 and TNF-α were significantly suppressed. Additionally, the hydrolysates showed strong antioxidant activity by scavenging DPPH and hydroxyl radicals and reducing intracellular reactive oxygen species, showing the ability to act simultaneously on oxidative stress, inflammation, and impaired re-epithelialization in diabetic wounds [57]. In an experimental model of infected wounds, Nuroini et al. (2025) evaluated the effects of oral royal jelly on epithelial regeneration in Pseudomonas aeruginosa-infected rats. Histological analysis conducted on day 14 revealed that treatment with 45 mg royal jelly increased the epithelial thickness to 43.21 μm, which was close to the normal tissue architecture, while treatment with 30 mg royal jelly increased the epithelial thickness to 38.05 μm, which was nearest to the amoxicillin-treated control group. The results suggest that royal jelly has a dose-dependent re-epithelialization effect and stimulates tissue regeneration in infected wounds [58].

The antimicrobial, antioxidant, and anti-inflammatory properties of royal jelly have also been extensively studied. Uthaibutta et al. (2023) tested royal jelly from Northern Thailand against Cutibacterium acnes, methicillin-resistant Staphylococcus aureus (MRSA), Staphylococcus aureus, Staphylococcus epidermidis, Pseudomonas aeruginosa and Corynebacterium spp., all skin pathogens. The antibacterial activity of royal jelly was significant for Gram-positive bacteria, with samples from Chiang Mai and Lamphun demonstrating quick bactericidal effects against *C. acnes*, MRSA, and *S. aureus*. Macrophage cultures showed that royal jelly could significantly decrease NO production and decrease the expression of iNOS in the cell culture and had antioxidant activity with a high phenolic and flavonoid content. The synergistic antimicrobial, antioxidant and anti-inflammatory activities make it a promising natural therapeutic agent to treat infected wounds [59]. Tsai et al. (2025) also confirmed the therapeutic effect of royal jelly-derived EVs for diabetic wound healing in streptozotocin-induced diabetic porcine models in recent years. The average particle size of the RJEVs was about 118 nm, which significantly enhanced the migration of fibroblasts by 31.4 ± 4.4%, restored the expression of collagen type I by 41.9 ± 7.6%, enhanced the migration of endothelial cells by 35.7 ± 5.6%, increased tube formation by 36.1 ± 2.4%, and upregulated the expression of the angiogenic markers VEGF and CD31 under hyperglycaemic conditions. In addition, RJEVs significantly downregulated the levels of pro-inflammatory cytokines (IL-1β, IL-6, IL-8, and TNF-α). They significantly enhanced wound healing in diabetic rats by 32.5 ± 7.2%, which revealed their capability to orchestrate wound resolution in inflammatory, fibroblast, collagen and angiogenic processes [60]. Together, these results provide substantial evidence that royal jelly affects the wound healing process by several complementary mechanisms: anti-oxidative stress and anti-chronic inflammation, stimulating keratinocyte and fibroblast migration, enhancing collagen deposition and angiogenesis, modulating immune responses, and strong antimicrobial activity. The multifunctional biological activities combined with recent studies revealing the extracellular vesicles of royal jelly as possible bioactive nanotherapeutics make royal jelly a promising natural source to be integrated into innovative wound dressings and nanotherapeutics-based therapeutic platforms for diabetic wound healing therapy.

### 3.4. Beeswax

Beeswax is secreted from the abdominal glands of the working honeybee and is used mainly in constructing the honeycomb [61]. It is mainly comprised of long-chain esters, hydrocarbons, free fatty acids, fatty alcohols and small quantities of aromatic compounds. Beeswax has relatively low microbial activity among other bee products like honey or propolis but is found in wound care due to its high biocompatibility, hydrophobicity, moisture retention, mechanical strength and film-forming abilities [62]. These properties add to the structural integrity of hydrogels, films, ointments, electrospun nanofibres, and composite scaffolds, control moisture loss, improve barrier properties, and facilitate controlled release of drugs, leading to an ideal microenvironment for tissue regeneration [63]. In addition to its structural function, beeswax also has biological properties that are beneficial in terms of tissue repair. Giampieri et al. (2018) showed that the byproducts of the beeswax recycling process (MUD1), rich in phenolic molecules, were able to activate human dermal fibroblasts against oxidative stress induced by AAPH. MUD1 at both doses of 250 and 750 μg/mL significantly decreased the intracellular accumulation of ROS and nitrite, reduced apoptosis, restored the levels of GSH, decreased the levels of lipid peroxidation and protein carbonyl, and increased the activity of the endogenous antioxidant enzymes (SOD, catalase, GPx, GR and GST). In addition, MUD1 enhanced mitochondrial respiration and returned its maximal respiratory capacity to AAPH-treated fibroblasts. Importantly, pre-treatment with MUD1 slightly improved scratch wound closure under normal conditions at both concentrations used (250 and 750 μg/mL), while AAPH significantly impaired the migration of fibroblasts and wound closure. This impairment was reversed by pre-treatment with 750 μg/mL MUD1, which restored the migration of the fibroblasts and wound closure to levels similar to the untreated control cells. These results suggest that the bioactive compounds obtained from beeswax have positive effects on tissue repair by preventing the damaging effects of oxidants on fibroblasts and improving cell migration, which is essential for wound re-epithelialization and tissue regeneration [64].

### 3.5. Bee Venom

Bee venom is a biologically complex secretion of a bee sting, which contains various low-molecular-weight compounds and biologically active peptides with different pharmacological activities [65]. It contains a number of major components such as melittin, apamin, phospholipase A_2_, hyaluronidase, adolapin, mast cell degranulation peptide and some bioactive amines. Of these, melittin is the main therapeutic peptide with strong antimicrobial, anti-inflammatory, analgesic, antioxidant and immunomodulatory activity [66]. At specific doses, bee venom has been shown to suppress inflammatory cytokines, inhibit the growth of microorganisms, stimulate angiogenesis, increase collagen production, and stimulate tissue regeneration. However, because of its potential to induce cytotoxicity, allergic reactions, and hypersensitivity, the therapeutic application of bee venom requires appropriate formulation strategies and precise dose optimisation to ensure safety and efficacy [67]. Bee venom’s therapeutic use in diabetic wound healing has been investigated at the molecular level. Badr et al. (2016) showed that topical bee venom significantly promoted wound healing in diabetic type I mice through the inhibition of oxidative stress caused by the activation of transcription factor-3 (ATF-3) and inducible nitric oxide synthase (iNOS). Diabetic wounds were characterised by decreased collagen synthesis, persistent inflammation, increased production of free radicals, upregulation of caspase-3, -8 and -9, and decreased expression of transforming growth factor-β (TGF-β) and vascular endothelial growth factor (VEGF), which resulted in impaired endothelial progenitor cell (EPC) mobilisation and defective angiogenesis. Bee venom treatment restored collagen production, normalised inflammatory cytokine levels, reduced oxidative stress, increased TGF-β and VEGF expression, inhibited apoptosis, and promoted mobilisation of long-lived EPCs, thereby significantly improving neovascularisation and wound healing in diabetic mice [68]. Mechanistic evidence was further provided by Hozzein et al. (2018), who reported that bee venom improved diabetic wound healing through restoring the Nrf2/Ang-1/Tie-2 signalling pathway. Collagen and β-defensin-2 (BD-2) expression in diabetic mice was significantly lower. Their macrophage and neutrophil recruitment capacity was impaired, ROS levels were high, and their antioxidant enzyme activity was low, while Tie-2 and its downstream signalling molecules eNOS, AKT and ERK were less phosphorylated, and expression of Ang-1 (angiopoietin-1) and Nrf2 (nuclear factor erythroid 2-related factor 2) was decreased. Bee venom treatment significantly increased collagen and BD-2 expression, restored antioxidant enzyme activities, enhanced Ang-1 and Nrf2 expression, activated Tie-2 downstream signalling, increased chemokine production (CCL2, CCL3, and CXCL2), rescued wound macrophages from mitochondrial apoptosis, and promoted CD31-positive neovascularisation, collectively leading to accelerated wound closure and enhanced tissue regeneration [69].

The effectiveness of bee venom has also been demonstrated in diabetic rat models. Namjou et al. (2022) compared the topical (0.25% bee venom ointment) and subcutaneous (0.5 mg/kg b.w.) routes in 60 alloxan-induced diabetic Wistar rats for 21 days. Bee venom treatment resulted in a significantly faster wound healing rate and lower serum glucose and triglyceride levels than diabetic control groups without bee venom treatment. Histopathological findings showed that bee venom-treated animals showed an improvement in the process of epithelial regeneration, collagen deposition, and granulation tissue formation. The use of systemic administration, however, was correlated with high levels of AST, ALT and urea in the serum, suggesting hepatotoxicity and renal toxicity, as well as the need to use localised topical administration and even optimisation of drug dosage [70]. In addition to its regenerative activity, bee venom also possesses strong antimicrobial properties, mostly through melittin. Bevalian et al. (2021) examined the antibacterial activity of melittin against vancomycin-resistant Staphylococcus aureus (VRSA) in a mouse model of third-degree burn infection. The minimum inhibitory concentration (MIC) and the minimum bactericidal concentration (MBC) for melittin against all VRSA isolates were found to be 0.125–2 and 0.125–4 μg/mL, respectively, showing remarkably low values. Mechanistic studies confirmed that melittin was membranolytic and was shown to provide rapid killing of bacteria, and a single topical application of 16 μg of melittin resulted in complete clearance of bacteria colonised with VRSA from burn wounds. Dermal toxicity and in vivo haemolysis were not observed, and thus melittin is a potential topical antimicrobial drug for wound management [71].

In recent years, bee venom has also been used in a variety of new therapeutic applications, using nano-enabled delivery systems. Celebi et al. (2026) studied bee venom-derived exosomes (BVE) in combination with metronidazole against Campylobacter jejuni. The combination of the two treatments showed an additive effect with an inhibition zone of 20 mm and 82.6% biofilm inhibition. BVE greatly inhibited oxidative stress and IL-1β production but promoted the expression of IL-10 and the total antioxidant capacity in the fibroblast wound-healing model. Additionally, the treatment suppressed the pro-apoptotic gene BAX and stimulated the anti-apoptotic gene BCL-2 and promoted the survival and wound regeneration of the fibroblasts, indicating that bee venom-derived exosomes could be an emerging nanotherapeutic platform that regulates infection, inflammation, oxidative stress, and wound repair simultaneously [72]. All these studies collectively show that bee venom has multiple complementary effects on wound healing, such as strong antimicrobial properties, anti-oxidative stress and anti-inflammatory signalling, antioxidant defence, stimulation of collagen synthesis, stimulation of angiogenesis via VEGF, Ang-1/Tie-2 and Nrf2 signalling pathways, release of endothelial progenitor cells, and protection of macrophages from apoptosis. However, its therapeutic use requires careful consideration of dose, route, and systemic exposure. Namjou et al. (2022) reported improved wound healing with topical 0.25% bee venom ointment and subcutaneous 0.5 mg/kg administration, but systemic administration increased AST, ALT, and urea levels, suggesting potential hepatic and renal toxicity. Therefore, localised topical delivery and dose optimisation are important to minimise systemic exposure. Despite toxicity and hypersensitivity concerns, several recent advances in topical formulations, controlled delivery systems, and bee venom exosomes may improve localised delivery and safety, although further clinical validation is required.

### 3.6. Integrative Mechanistic Perspective of Beehive-Derived Products in Wound Healing

**Figure 3:**
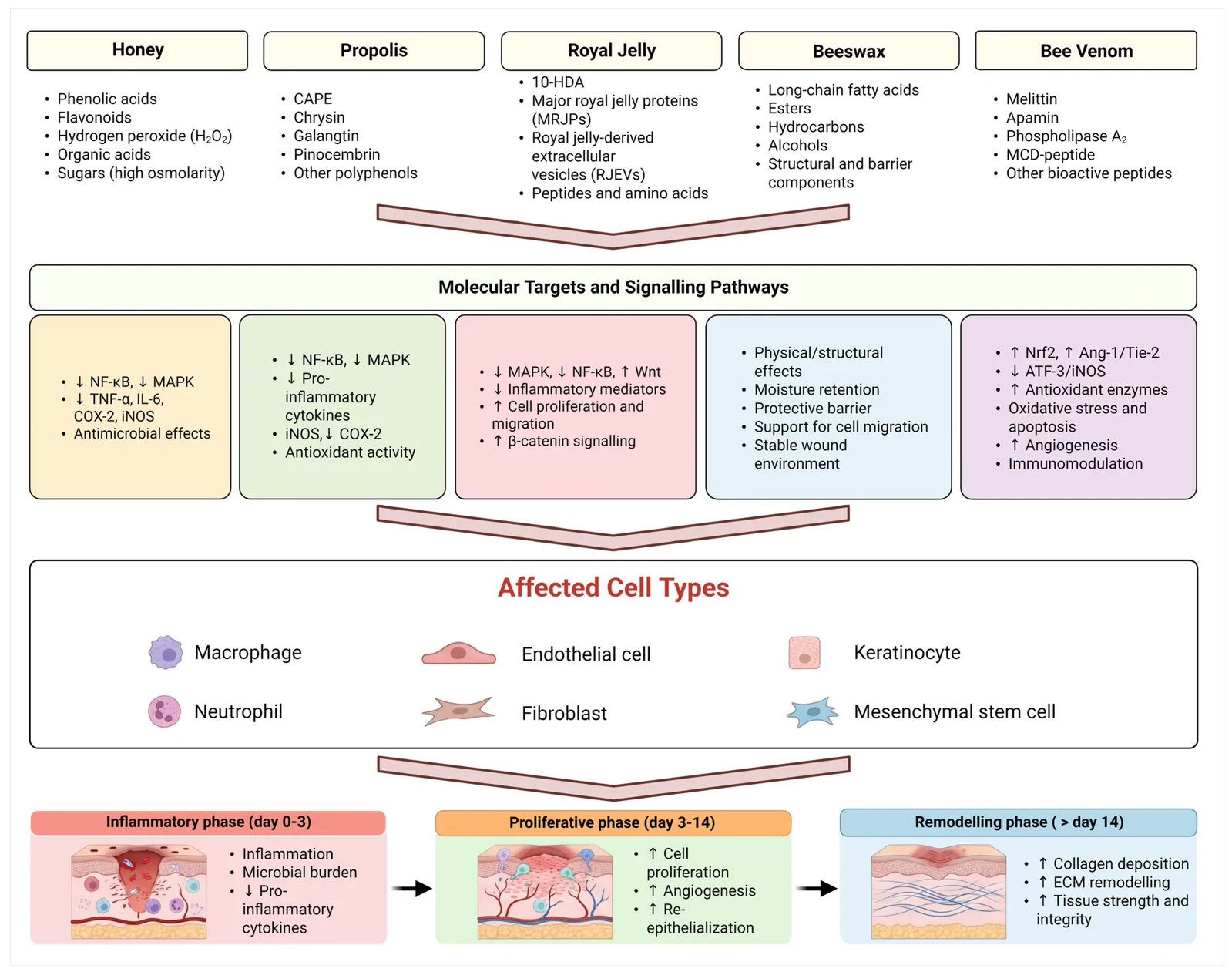
Molecular mechanisms of beehive products in diabetic wound healing, highlighting their bioactive components, molecular targets, affected cell types, and roles in inflammatory, proliferative, and remodeling phases. Created in BioRender. Ishaya, J. (2026) https://BioRender.com/c1n7a6s.

The wound-healing effects of beehive-derived products can be understood as interconnected regulation of inflammation, oxidative stress, antimicrobial defence, angiogenesis, cellular proliferation, and ECM remodelling rather than as independent molecular events [73]. During the inflammatory phase, bioactive constituents of honey, propolis and royal jelly can attenuate excessive inflammatory signalling, including NF-κB and MAPK pathways, thereby reducing mediators such as TNF-α, IL-6, iNOS and COX-2 [74]. In parallel, antioxidant mechanisms involving Nrf2 and endogenous antioxidant enzymes can reduce excessive reactive oxygen species, which is particularly relevant to the oxidative environment of diabetic wounds [75]. Bee venom has demonstrated complementary effects through suppression of ATF-3/iNOS-mediated oxidative stress and restoration of Nrf2/Ang-1/Tie-2 signalling, contributing to macrophage survival and angiogenesis [76]. During the proliferative phase, these anti-inflammatory and antioxidant effects are accompanied by enhanced keratinocyte and fibroblast migration, collagen deposition, VEGF-associated angiogenesis and endothelial progenitor-cell recruitment. Wnt-associated signalling observed with royal-jelly-derived formulations may further contribute to epidermal development and tissue repair [77]. Collectively, these mechanisms indicate that beehive-derived products may influence multiple interconnected stages of wound healing, from inflammatory resolution and oxidative-stress control to re-epithelialisation, neovascularisation and tissue remodelling (Figure 3).

Diabetic foot ulcers frequently harbour polymicrobial microbial communities and biofilms, which can reduce antimicrobial susceptibility and contribute to persistent infection. Therefore, the antibiofilm potential of beehive-derived products is particularly relevant to chronic wound management. Honey and bee-venom-derived melittin have demonstrated antimicrobial and antibiofilm activity, with some studies also indicating enhanced activity when combined with conventional antimicrobial agents [78]. However, such findings should be distinguished from confirmed synergistic interactions unless appropriate combination studies and synergy analyses have been performed. Similarly, evidence against planktonic bacteria should not be directly extrapolated to established biofilms, which require specific assessment using preformed biofilm models. Activity against resistant wound-associated organisms, including MRSA, and fungal co-infections such as Candida spp. also warrants consideration because these organisms may contribute to chronic wound persistence [79]. Overall, although the available evidence supports the potential of honey and melittin as adjunctive antimicrobial strategies, direct evidence in polymicrobial diabetic wound biofilms remains limited and requires further investigation.

## 4. Advanced Formulation Approaches

**Figure 4:**
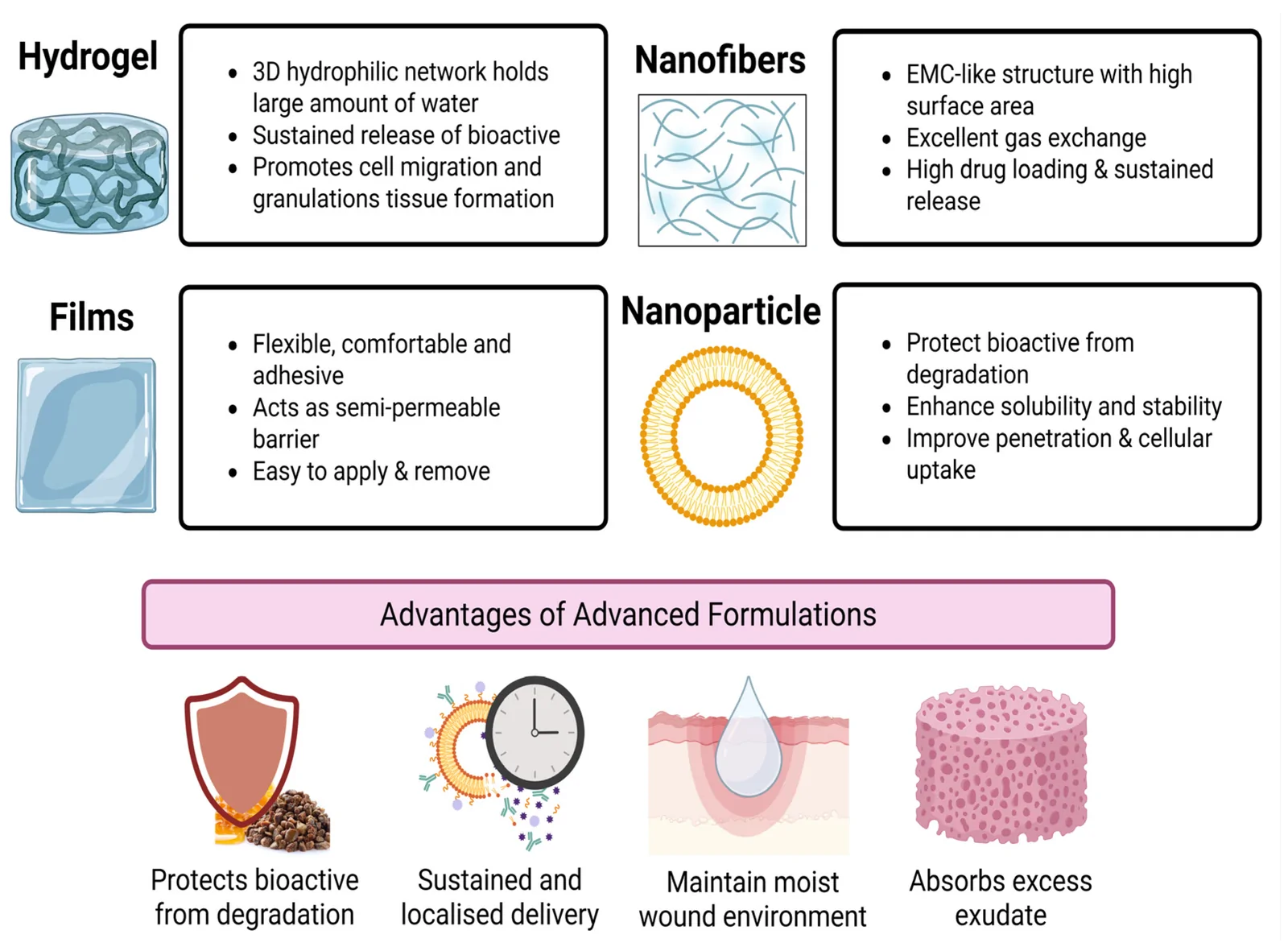
Advanced formulation approaches for beehive-derived products, including hydrogels, films, nanofibers, and nanoparticles, and their major advantages in wound management, such as protection of bioactives, sustained and localised delivery, moisture maintenance, and exudate absorption. Created in BioRender. Ishaya, J. (2026) https://BioRender.com/c1n7a6s.

The therapeutic benefits of bee-derived products in wound healing can be further enhanced by formulation approaches that can modulate the physicochemical stability, bioavailability, controlled release and in-wound retention of these products [80]. However, the direct use of honey, propolis, royal jelly, beeswax and bee venom is restricted due to their variable composition, limited stability, rapid dilution by wound exudate, and difficulty in maintaining adequate concentrations within the wound microenvironment [81]. Advanced wound-dressing platforms can address these limitations by protecting bioactive constituents from degradation, improving their retention at the wound site, and enabling sustained and localised delivery. These systems can also maintain an appropriate moist environment, facilitate gas exchange, absorb excess exudate, and provide mechanical protection, thereby improving the functional performance of beehive-derived products within the wound environment [82]. Thus, the incorporation of beehive products with natural and synthetic biomaterials has resulted in the creation of multifunctional dressings that can simultaneously control infection, inflammation and oxidative stress, promote angiogenesis, and increase tissue regeneration in chronic wounds (Figure 4). From a materials-science perspective, the selection of the delivery platform should be based on the physicochemical limitations of the native bee-derived product and the requirements of the wound. For example, hydrogels and films can improve wound-site retention and moisture management; nanofibrous systems can provide close wound-bed contact and a high surface area for bioactive delivery [83]. Nanoparticle-based systems can improve the dispersion, stability, and localised delivery of poorly soluble or unstable constituents. However, the biological activity of the carrier should also be considered when interpreting these findings, as some materials, such as chitosan, possess intrinsic antimicrobial and regenerative properties [84]. Therefore, improvements observed with composite formulations cannot be attributed exclusively to the beehive-derived product unless appropriate blank-carrier controls and comparative formulations are included. Similarly, enhanced effects should not be described as synergistic unless supported by suitable combination and dose-dependent studies [85].

The representative formulations of beehive products are summarised in Table 2 as both hydrogel and film formulations. The reported findings are critically interpreted by considering both the contribution of the beehive-derived bioactive and the potential biological effects of the carrier or dressing material.

### 4.1. Honey-Based Dressings

Honey-based dressings are among those formulations studied in detail in animal models for wound healing, as they benefit from the therapeutic properties of honey and the properties of modern biomaterials. Preliminary research indicates that dressings coated with honey have antimicrobial, antioxidant, anti-inflammatory properties and the ability to stimulate granulation tissue formation, collagen deposition, re-epithelialization and wound closure [99]. Honey has been added to hydrogels, films, foams, electrospun nanofibres and composite scaffolds made from biocompatible polymers like chitosan, polyvinyl alcohol (PVA), gelatin, alginate, hyaluronic acid and cellulose derivatives to improve their stability and therapeutic effects. The advanced delivery systems enhance the retention of honey, promote a moist environment of the wound, ensure the prolonged release of bioactive components and consequently improve overall wound healing in preclinical models [100]. Recent formulation studies have shown that the physicochemical and biological behaviour of conventional honey dressings is greatly enhanced by polymeric honey hydrogels. Chopra et al. (2022) used a solvent-casting method to produce honey-loaded chitosan/PVA hydrogel films and found that they had excellent mechanical and physicochemical properties for use in wound dressing. The water vapour transmission rate (WVTR) of the hydrogel films ranged from 1650.50 ± 35.86 to 2698.65 ± 76.29 g/m^2^/day, the tensile strength ranged from 4.74 ± 0.83 to 38.36 ± 5.39 N, and the elongation at break ranged from 30.58 ± 3.64 to 33.51 ± 2.47 mm. SEM analysis showed a smooth surface morphology, whereas FTIR and DSC analysis confirmed the successful incorporation of honey into the polymeric matrix. In vitro release studies showed good release of honey, and the hydrogel films exhibited significant antibacterial activity against Staphylococcus aureus, suggesting their use in multifunctional wound healing applications that can give prolonged therapeutic effects [101].

Furthermore, naturally derived polymeric hydrogels enriched with honey have been developed to further boost cellular compatibility and wound healing. This enhancement in cellular compatibility and wound repair was achieved by Şalva et al. (2023) by formulating chitosan/hyaluronic acid/honey hydrogels without the use of chemical crosslinking agents. The addition of honey decreased water absorption by 12%, decreased the porosity of the hydrogels from 17% to 12% and did not affect the interconnected porous structure. The incorporation of HA resulted in increased viscosity of the hydrogel, while honey slightly decreased it, which further enhanced the handling characteristics of the hydrogel. Specifically, the adhesion and proliferation of fibroblasts were 144% in honey-stimulating hydrogels, compared with the control hydrogels. In addition, in vivo wound healing assays revealed that honey incorporation accelerated wound closure, which corroborates the synergistic wound-healing effects of hyaluronic acid and honey in a biomimetic ECM [86].

Honey-based natural composite hydrogels also exhibited good antibacterial and regenerative properties. Zhang et al. (2022) prepared a composite hydrogel with Aloe polysaccharide (AP) and honey, which was obtained by cross-linking acetylated β-mannan isolated from Aloe barbadensis and PVA in the presence of honey. Such a hydrogel had very high mechanical strength, good blood compatibility and high cytocompatibility with NIH-3T3 and L929 fibroblast cells. Significantly, the composite hydrogel demonstrated wide-range antibacterial activity against Staphylococcus aureus, Escherichia coli and Candida albicans and enhanced healing of infected full-thickness wounds. The synergistic effect of aloe polysaccharide with honey demonstrated its ability to keep the wound moist, control microbial proliferation, and stimulate tissue regeneration, thus underlining the benefits of multifunctional natural-polymer composite wound dressings [87]. Zainuddin et al. (2025) prepared a Kelulut honey-doped gelatin/PVA crosslinked with 0.1% genipin as a biodegradable skin substitute in another study. The GE-PVA-H1-GNP (1% Kelulut honey) formulation showed the best physicochemical properties: swelling ratio (86.31 ± 14.27%) water vapour transmission rate (501.21 ± 41.35 g/m^2^/day), biodegradation rate (0.0036 ± 0.0003 g/h), contact angle (38.46° ± 3.89°), resilience (98.85 ± 1.03%) and compression strength (77.42 ± 7.17%). MTT assays showed excellent cytocompatibility in all formulations of the hydrogel, with good mechanical stability, hydrophilicity and biocompatibility for the use of a low concentration of Kelulut honey in the hydrogel for cutaneous tissue regeneration [88]. These studies showed that the addition of honey to advanced hydrogel and composite biomaterial systems significantly improved the mechanical stability, moisture control and maintenance of bioactive release, antimicrobial properties, and cellular compatibility, which in turn significantly increased wound healing potential. Honey can be blended with natural and synthetic polymers, including chitosan, hyaluronic acid, aloe polysaccharides, gelatin, and PVA, to create wound dressings with multipurpose properties, similar to the ECM, which enhance the proliferation of fibroblasts, a moist environment, and regeneration of the tissue. The results indicate that the dressings, which are made from honey, could be a new-generation therapeutic platform for the effective treatment of chronic diabetic wounds.

### 4.2. Hydrogel- and Film-Based Formulations

Hydrogels and film-based wound dressings have been developed as versatile platforms for delivering bioactives derived from a beehive, due to their high water content and excellent biocompatibility, as well as their structural properties that closely mimic the ECM [102]. The improvement of the physicochemical stability of natural products, sustained and localised release of bioactive components and increased therapeutic efficacy are achieved by incorporation into polymeric hydrogels and films of honey, propolis, royal jelly, beeswax and bee venom [103]. Numerous polymers, both natural and synthetic, such as chitosan, PVA, gelatin, alginate, hyaluronic acid, pullulan, pectin, methylcellulose and silk fibroin, have been used successfully to prepare such formulations [104]. Preclinical studies have shown that hydrogel- and film-based dressings have excellent mechanical properties, moisture-holding capacity, antimicrobial and antioxidant effects, along with the stimulation of fibroblast proliferation, collagen deposition, angiogenesis and accelerated wound healing [105].

### 4.3. Nanoengineered Delivery Systems

Nanoengineered delivery systems have emerged as novel platforms for enhancing the stability, bioavailability and therapeutic efficacy of bioactive compounds derived from the beehive in wound healing [106]. For sensitive bioactive properties, nanoparticles, liposomes, nanoemulsions and electrospun nanofibers help to shield the properties from degradation, ensure local and sustained release of drug molecules, promote enhanced cellular uptake, and extend the time spent at the wound site [107]. Of these, the three-dimensional structure of electrospun nanofibers closely resembles that of the original ECM and offers great potential for cell adhesion, proliferation, migration, and tissue regeneration, along with adequate gas exchange and moisture balance [108]. The addition of honey, propolis, royal jelly, beeswax, and bee venom to the nanoengineered platforms has proven to enhance antimicrobial, antioxidant, and anti-inflammatory properties, which boost wound healing and tissue repair. The recent study has provided evidence for the skin regeneration potential and use of propolis-loaded electrospun nanofibers. Doodmani et al. (2024) fabricated nanofiber-nets (PCL/COL) with 5%, 10% and 15% propolis extract by electrospinning-netting technology. The scaffolds were composed of nanofibers of the trunks ranging from 164–728 nm in diameter, connected to nanonets with about 20 nm diameter, similar to those of the native ECM. The water vapour transmission rates of the nanofibers loaded with propolis were similar to those of the commercial Tegaderm dressings, and the cytoprotective activity of the propolis-loaded nanofibers was dose-dependent, showing the highest HFF-2 fibroblast viability when exposed to nanofibers containing 15% propolis after 24, 48, and 72 h. Furthermore, the scaffolds showed excellent hemocompatibility (<3% hemolysis), making them suitable for wound healing applications [109]. Similarly, Yu et al. (2025) prepared PVA/chitosan electrospun nanofibres loaded with royal jelly, which had an average diameter of 219–299 nm. The nanofibers showed a high encapsulation efficiency (89.0%) and cumulative release (84.87%), along with very good water vapour permeability and hydrophilic properties. The optimised ratio of PVA/chitosan of 7:3 exhibited the best antibacterial activity, with inhibition rates of 97.83% against *S. aureus* and 72.08% against *E. coli*, respectively, indicating that the nanofibers loaded with royal jelly have great potential as a multifunctional wound dressing material [110].

Nanoengineered systems have also been investigated for delivering bee venom to enhance antimicrobial activity with a reduced level of toxicity. Aburayan et al. (2022) developed electrospun PVP fibres loaded with the antimicrobial peptide melittin as antimicrobial wound patches, with an average fibre diameter of 1030 ± 160 nm, drug loading of 49 ± 3 μg/mg, and entrapment efficiency of 84 ± 5%. The formulation exhibited approximately 40% melittin release within 5 min and complete release within 120 min, while maintaining low cytotoxicity toward human dermal fibroblasts at concentrations ≤10 μg/mL. The minimum inhibitory concentration (MIC) was 5 μg/mL against *S. aureus*, *A. baumannii*, *E. coli*, and *C. albicans*, and nanoencapsulation did not significantly affect the zone of inhibition [111]. Beeswax/poly-L-lactic acid (PLA) electrospun membranes were produced by Zou et al. (2020) that possessed a superhydrophobic surface without destroying the porous fibrous structure. After 7 days of culture, the membranes significantly suppressed fibroblast proliferation, while peritendinous adhesion formation was significantly suppressed in a rat Achilles tendon model, compared with PLA membranes alone, indicating the usefulness of electrospun membranes with beeswax as an anti-adhesion barrier for tissue repair [112]. These studies provide proof that nanoengineered delivery systems significantly improve the therapeutic efficiency of beehive-derived bioactive compounds in terms of the ability to encapsulate the drug, controlled drug release, mechanical stability, antimicrobial activity, and cell compatibility. Nanostructured platforms such as electrospun nanofibers and biomimetic architectures show the potential for the highest biological efficacy of honey, propolis, royal jelly, beeswax, bee venom, etc. to become next-generation wound dressing systems in chronic and diabetic wound management.

### 4.4. Composite and Biopolymer-Based Dressings

Composite and biopolymer-based dressings combine bioactive compounds present in the beehive with natural biomaterials to promote wound healing, along with desired structural integrity and drug delivery [113]. The scaffolds for tissue regeneration are excellent in terms of biocompatibility, biodegradability, moisture retention and mechanical stability. These biopolymers include collagen, chitosan, gelatin, silk fibroin, sericin, alginate, PVA, and acellular extracellular matrices [114]. The addition of these matrices with honey, propolis, royal jelly and bee venom helps better stabilise and slowly release the bioactive compounds and promotes antimicrobial, antioxidant, anti-inflammatory and regenerative properties [75]. These composite dressings have been shown to have the ability to stimulate the proliferation and migration of fibroblasts, induce angiogenesis and collagen deposition, modulate inflammatory response and accelerate wound healing in the preclinical setting, which makes them promising multifunctional biomaterials to be used in chronic and diabetic wound management. Recent developments have emphasised the development of propolis with naturally derived ECM scaffolds in order to enhance the antimicrobial and regenerative properties. To create a propolis-functionalized acellular fish skin (EPE-AFS) scaffold, Bakhtazad et al. (2026) used the vacuum-assisted impregnation approach. The scaffold maintained native collagen architecture and showed an improvement in the water contact angle, which dropped from 91 ± 2° to 67 ± 3° (more hydrophilic) and retained a mechanical strength of around 13 MPa. The formulation released >90% of the polyphenol content within 96 h, promoted cell adhesion and migration of fibroblasts, increased expression of regenerative markers such as COL1A1, COL3A1, VEGF and TGF-β1, decreased expression of inflammatory markers such as IL-6 and MMP-9, and inhibited the growth of Staphylococcus aureus and Escherichia coli. The results show that collagen in a natural scaffold could be used as a sustained-release vehicle for propolis in wounds [115].

Similarly, Tan et al. (2023) developed an in situ photo-crosslinked sericin methacrylate (SerMA) hydrogel incorporating royal jelly-derived extracellular vesicles (RJ-EVs) to simultaneously modulate inflammation and promote vascular regeneration. The porous hydrogel allowed for a slow release of RJ-EVs, with high fluid absorption and excellent tissue conformity. The antioxidant and anti-inflammatory activities were observed to be quite substantial in in vitro studies, which showed significant enhancement in proliferation and migration of the L929 fibroblast cell line. The SerMA/RJ-EVs hydrogel promoted a healing rate of 96.8% for a full-thickness skin wound model, together with improvement in angiogenesis and cell proliferation. Further, RNA sequencing identified activation of pathways linked to epidermal development, Wnt signalling, and inflammatory tissue repair, further illustrating the multifunctional regenerative capacity of this bioactive composite dressing. These studies show that composite and biopolymer-based dressings are effective approaches in the integration of therapeutic benefits of beehive products and structural benefits of natural biomaterials. These multifunctional systems, equipped with bioactive compounds and ECM-mimicking scaffolds, not only boost antimicrobial efficacy and control inflammation but also promote angiogenesis and collagen remodelling while dramatically accelerating tissue regeneration, which makes them ideal for future generation of wound dressings [116].

### 4.5. Platform Selection According to Wound Characteristics

The selection of an appropriate delivery platform should be guided by the specific characteristics of the diabetic wound. Hydrogels and hydrogel films may be particularly suitable for dry wounds because they help maintain moisture and provide prolonged contact with the wound bed, whereas highly exuding wounds may require absorbent composite or sponge-like systems capable of managing excess wound fluid while maintaining structural integrity [117,118]. In infected wounds, antimicrobial platforms such as honey-, propolis-, or bee-venom-loaded hydrogels, films, and nanofibres may provide localised antimicrobial activity together with sustained delivery. For wounds characterised by excessive inflammation and oxidative stress, hydrogels, nanofibres, and nanoengineered systems may facilitate localised and prolonged delivery of antioxidant and anti-inflammatory beehive-derived bioactives [119]. For fragile or granulating wounds, soft, non-adherent hydrogels and nanofibrous dressings may be preferable because they can protect the wound bed and minimise trauma during dressing removal. Thus, no single formulation platform is universally appropriate for diabetic wounds, and selection should consider moisture balance, exudate burden, infection, tissue fragility, bioactive retention, and the required dressing or dosing interval.

However, evidence regarding the in vivo bioavailability, tissue penetration, stability, and pharmacokinetic behaviour of individual bee-derived bioactive compounds remains limited. Advanced delivery systems such as hydrogels, nanofibres, nanoparticles, and composite scaffolds may improve local retention, protect unstable constituents, and enhance penetration into the wound bed. However, the therapeutic concentrations achieved at the target tissue and the systemic exposure of individual bioactive constituents have not been adequately established [120]. Therefore, future studies should evaluate in vivo drug release, tissue distribution, local bioavailability, stability, and pharmacokinetic profiles using standardised formulations.

## 5. Clinical Studies

Although substantial evidence supporting the wound-healing potential of beehive products has been generated from preclinical investigations, clinical studies remain relatively limited. The clinical evidence available is on the use of honey, propolis, formulations containing beeswax, and bee venom, and most of the studies report better wound healing, wound pain reduction, antimicrobial activity, and safety profiles. However, the majority of these investigations consist of case reports, pilot studies, or small randomised clinical trials, emphasising the need for larger multicenter studies to validate their therapeutic efficacy. Honey is one of the most promising beehive products for treating chronic diabetic wounds. Indonesian randu honey was successfully used as a dressing material in a 54-year-old patient with uncontrolled type 2 diabetes mellitus with an extensive chronic lower-limb wound with bone, tendon, muscle and meniscus exposure who was initially recommended for amputation [121]. After serial debridement, split-thickness skin grafting, insulin therapy and vacuum-assisted closure of the wound, and regular application of topical honey, the wound healed after about 1.5 years, and the patient was able to walk with a walking stick. Significantly, there was no allergic reaction, no secondary infections, and no adverse effects of the treatment observed, which showed that the Indonesian randu honey is safe and effective for use as an adjunct in the treatment of complex diabetic wounds at a low cost.

Clinical evidence has also shown that propolis proved to be effective in postoperative wound management. Alizadeh Tabari et al. [122] carried out a split-mouth, double-blind randomised clinical trial with 10 patients who were undergoing harvesting of free gingival grafts on either side of the mouth. Propolis 5% oral ointment significantly reduced donor site pain on day 1 and day 3 as compared with placebo. In addition, the colour matching of the wound was improved significantly on days 14, 21, 30 and 42, showing that wound healing is improved. There was no difference in bleeding, tissue thickness or burning sensation, however, indicating that propolis has greater effects on patient comfort and soft tissue healing in the initial healing phase. For beeswax-based products only, Modarresy et al. assessed the effectiveness of a topical product called RepoGen cream, comprising lanolin, beeswax, and olive oil, in a double blind, randomised, placebo-controlled study of 133 breastfeeding women (63 intervention, 70 placebo). The intervention also had a significant effect on reducing nipple pain on day 3 (63.5% vs. 42.9%) and on day 7 (84.1% vs. 71.4%), and the incidence of nipple wounds, redness and bleeding was also reduced. Importantly, there were no reported maternal or neonatal adverse effects, thus confirming the safety and clinical efficacy of the beeswax-based topical formulations in the repair of epithelia [123].

Bee venom has also demonstrated positive clinical outcomes. Othman et al. conducted a single-blind randomised controlled trial with 100 patients suffering from chronic diabetic foot ulcers or venous leg ulcers and randomly divided them into a bee venom phonophoresis or control group. The group treated with bee venom gel + therapeutic ultrasound showed significantly better wound surface area and ulcer volume reduction after 6 and 12 weeks when compared with conventional therapy alone. A further histopathological evaluation also showed significantly higher expression of the Ki-67 proliferation marker, which correlates with more proliferation and tissue regeneration. Both diabetic and venous ulcers responded well, with a greater response in venous ulcers, which demonstrates the efficacy of bee venom phonophoresis as an adjunctive treatment for chronic wound healing [124]. There are several registered clinical studies evaluating the therapeutic effects of beehive product-based interventions on the treatment of acute, chronic and diabetic wounds, mainly involving honey and propolis. These studies included pilot studies and randomised controlled studies and included outcome measures including wound healing, epithelialization, infection control and scar formation (Table 3).

Despite encouraging clinical findings, the strength of evidence remains limited by small sample sizes, heterogeneous interventions, variable wound types, and methodological limitations. A recent 2026 systematic review and meta-analysis of 16 RCTs involving 1423 participants reported a higher complete-healing rate with honey dressings (OR 2.28, 95% CI 1.76–2.95) and reduced healing time (MD −4.38 days, 95% CI −8.06 to −0.71); however, the certainty of evidence was rated moderate for healing rate and low for healing time because of methodological limitations [125]. Additionally, the IWGDF 2023 guideline update recommends against the use of honey or other bee-derived products specifically to promote healing in diabetes-related foot ulcers, as the available randomised controlled trials were considered to have a high risk of bias and the certainty of evidence for benefit was very low [126]. Thus, although recent meta-analytic evidence suggests a potential benefit of honey dressings, differences in study quality, wound characteristics, interventions, and methodological rigour limit definitive conclusions regarding their efficacy in diabetic foot ulcers. In another double-blind, placebo-controlled clinical trial involving 64 ulcers, topical application of 5% royal jelly did not produce statistically significant differences compared with placebo in ulcer depth, length, or width reduction, healing duration, or complete healing [127]. These findings indicate that the promising wound-healing effects observed in preclinical studies cannot be directly extrapolated to clinical efficacy. Therefore, further adequately powered clinical trials are required to establish the therapeutic effectiveness of topical royal jelly in chronic and diabetic wounds.

## 6. Challenges and Future Perspectives

Although beehive products have potential therapeutic applications, there are a number of barriers to wider use in wound management. The composition and activity of honeys, propolis, royal jelly, and other bee products can vary significantly depending on the variety of origin, geographic location, bee species, season, processing and storage conditions. The variability complicates the standardisation, reproducibility and dose optimisation. Moreover, certain active ingredients exhibit poor aqueous solubility, low stability, and rapid degradation or dilution in wound exudate, which may decrease their therapeutic efficacy. These limitations can be addressed by the use of advanced delivery systems, e.g., hydrogels, films, nanofibres, nanoparticles and composite scaffolds with enhanced stability, local retention and controlled release. Safety and tolerability also require careful consideration, particularly for bee venom. Bee venom and its elements have antimicrobial, anti-inflammatory, antioxidant and regenerative properties, but may be cytotoxic and/or cause hypersensitivity or systemic toxicity if inappropriate concentrations are used. Hence, dose optimisation, localised delivery and systemic exposure-minimising formulation should be targeted in future studies. In addition, clinical evidence is also rather limited compared with preclinical evidence. Current clinical research consists of case reports, pilot studies and small randomised trials, which warrant further investigation in large, well-designed, multicentric trials that use standardised formulations and clinically relevant endpoints. Safety concerns should also be considered for honey, propolis, and royal jelly, particularly because these products may cause allergic or hypersensitivity reactions in susceptible individuals. Propolis may cause contact dermatitis, while honey and royal jelly may cause allergic reactions, particularly in individuals sensitized to bee products or pollen. Therefore, patient screening for previous allergy or hypersensitivity to bee products, pollen, or related ingredients should be considered before topical application. Patch testing may be considered in individuals with a relevant allergy history, and treatment should be discontinued if allergic reactions occur. Standardized preparations with appropriate quality and safety testing are also important to minimize variability and potential adverse effects. The variability in therapeutic outcomes may also arise from differences in the chemical composition of beehive-derived products. Botanical and geographical origin can influence the concentration and profile of phenolics, flavonoids, peptides, enzymes, and other bioactive constituents, while bee species, environmental conditions, season, harvesting practices, and storage can further affect their composition and biological activity. Processing methods such as heating, drying, extraction, and filtration may additionally alter heat-sensitive or volatile constituents. These factors can contribute to differences in antimicrobial, antioxidant, anti-inflammatory, and regenerative effects reported across studies and may limit reproducibility between batches and experimental models. Therefore, standardized sourcing, processing, chemical characterization, and quality-control procedures are essential for improving consistency and facilitating comparison across studies.

Future studies should focus on the preparation of standardised medical-grade preparations with precisely defined chemical composition, consistent biological activity and well-defined quality control criteria. Greater emphasis should also be placed on understanding the molecular mechanisms underlying the synergistic actions of different bee products and their combinations with biomaterials. New delivery platforms such as extracellular vesicle-based systems and multifunctional composite scaffolds are emerging as a new frontier for the evolution of controlled delivery and targeting multiple routes in one delivery vehicle. Additionally, translation to the clinic will involve long-term safety, stability, manufacturing scalability, sterilisation, storage, cost-effectiveness and regulatory considerations. Economic and practical feasibility should also be considered for clinical translation. Compared with some conventional advanced wound-care products, locally sourced bee-derived products may offer potential affordability advantages, particularly when standardized production and simple delivery systems are used. Further clinical trials on beehive-based treatments would be beneficial in testing them against the current accepted standard of care in various types of wounds, such as diabetic, venous, pressure, burn, traumatic, and post-surgical wounds. These will help to establish what products and formulations are most therapeutically beneficial and their place in contemporary wound management. To facilitate the translation of beehive-derived advanced dressings from laboratory research to clinical practice, their development can be considered across three sequential translational levels. Level I—Biological validation should establish the biological activity, mechanisms of action, antimicrobial and antibiofilm effects, antioxidant and anti-inflammatory responses, dose–response relationships, and reproducibility using relevant wound models. Level II—Preclinical engineering validation should evaluate formulation stability, mechanical properties, controlled release, biocompatibility, sterilisation compatibility, degradation behaviour, scalability, and performance in clinically relevant diabetic wound models. Level III—Clinical translation should establish standardised medical-grade preparations, reproducible manufacturing processes, regulatory compliance, safety, and therapeutic efficacy through adequately powered clinical trials using standardised and clinically relevant endpoints.

## 7. Conclusions

Beehive-derived products represent promising multifunctional therapeutics for the management of acute and chronic wounds, including diabetic foot ulcers, burn wounds, venous ulcers, pressure wounds, traumatic injuries, and post-surgical wounds. Honey, propolis, royal jelly, beeswax, and bee venom contain diverse bioactive constituents that collectively exhibit antimicrobial, antioxidant, anti-inflammatory, immunomodulatory, and regenerative activities. These properties enable them to act on several interconnected pathological processes responsible for delayed wound healing, including persistent inflammation, oxidative stress, microbial infection, impaired angiogenesis, defective collagen deposition, and extracellular matrix remodelling. The incorporation of these products into hydrogels, films, electrospun nanofibres, nanoparticles, and composite scaffolds has further enhanced their stability, local retention, controlled release, and therapeutic performance. Preclinical findings demonstrate their ability to promote fibroblast proliferation, re-epithelialisation, collagen formation, angiogenesis, microbial control, and wound closure. Clinical investigations have also reported encouraging outcomes, particularly for honey and propolis, while beeswax- and bee venom-based interventions have shown potential in specific wound conditions. However, clinical evidence remains limited and is frequently based on small trials or pilot studies, highlighting the need for larger, well-controlled studies.

## Figures and Tables

**Table 1:** Evidence categorization of beehive-derived products across wound models and their relevance to diabetic wound healing.

Evidence Category	Model/Wound Type	Examples in Manuscript	Relevance to Diabetic Wounds	Interpretation
Direct diabetic evidence	Diabetic animal models	Honey, royal jelly, bee venom	High	Primary evidence
Direct diabetic cellular evidence	Hyperglycaemic/AGE-damaged cells	Royal jelly hydrolysates, RJ-derived systems	High–moderate	Mechanistically relevant
Diabetic clinical evidence	DFU/diabetes-associated wounds	Honey, bee venom interventions	Highest clinical relevance	Clinical evidence
Chronic non-diabetic wounds	Venous ulcers, chronic infected wounds	Bee venom and honey studies	Moderate	Supportive/extrapolated
Acute animal wounds	Excision/incision/full-thickness wounds in healthy animals	Propolis, honey, RJ formulations	Limited–moderate	Mechanistic/formulation evidence
Burn wounds	Burn/infected burn models	Propolis, melittin	Limited	Antimicrobial/regenerative support
Surgical/postoperative wounds	Oral donor sites, surgical wounds	Propolis/honey	Limited	Extrapolated
Other wound indications	Nipple fissures, tendon injury	Beeswax-containing formulations	Low for DFU	Extrapolated

**Table 2:** Representative preclinical hydrogel, film, and composite formulations of honey, propolis, royal jelly, beeswax, and bee venom for wound healing applications.

Bee Product	Formulation/Dressing	Polymer/Carrier	Key Findings	Major Outcome/Application	Ref
Honey	Hydrogel	Chitosan/Hyaluronic acid	Porosity reduced from 17% to 12% after honey incorporation; fibroblast proliferation increased to 144%; accelerated wound healing in vivo.	Honey enhanced cell adhesion, proliferation, and tissue regeneration without chemical crosslinkers.	[86]
Honey	Composite hydrogel	Aloe polysaccharide/PVA	Excellent blood compatibility and fibroblast cytocompatibility; antibacterial against *S. aureus, E. coli,* and *C. albicans*; accelerated infected wound healing.	Multifunctional hydrogel combining antibacterial activity with enhanced tissue regeneration.	[87]
Kelulut honey	Hydrogel	Gelatin/PVA + 0.1% Genipin	Optimal formulation (GE-PVA-H1-GNP): swelling 86.31 ± 14.27%, WVTR 501.21 ± 41.35 g/m^2^/day, biodegradation 0.0036 ± 0.0003 g/h, contact angle 38.46 ± 3.89°, resilience 98.85 ± 1.03%, compression strength 77.42 ± 7.17%.	Mechanically robust, biodegradable, and highly biocompatible skin substitute.	[88]
Propolis	Hydrogel	PVA	Polyphenols 34.82 ± 0.08 mg GAE/g; flavonoids 23.61 ± 0.05 mg QE/g; burn wound contraction 93.58 ± 0.15%; excision wound contraction 91.45 ± 0.29%; *p* < 0.0001.	Accelerated wound contraction and re-epithelialization comparable to povidone-iodine ointment.	[89]
Propolis	Hydrogel film	Quaternized chitosan/Pectin	Improved tensile strength and stability; antioxidant activity 21–36%; controlled propolis release; antibacterial against *S. aureus* and *S. pyogenes*; non-toxic to fibroblasts.	Controlled-release hydrogel with antioxidant, antibacterial, and wound-healing properties.	[90]
Propolis	Hydrogel	Polyacrylamide/Methylcellulose	Polyphenols: 24.74 ± 1.71–32.10 ± 1.01 mg GAE/g; flavonoids: 8.01 ± 0.99–13.81 ± 0.71 mg QE/g; excellent swelling, antioxidant activity (DPPH, ABTS, FRAP), and biocompatibility.	Promising antioxidant wound dressing with desirable mechanical and rheological properties.	[91]
Propolis	Electrospun nanofibers	Silk fibroin/Gelatin	1% propolis formulation showed excellent cytocompatibility and hemocompatibility; enhanced L929 fibroblast migration; antibacterial against *S. aureus* and *E. coli*; accelerated healing in full-thickness wounds.	Bioactive nanofibrous dressing mimicking ECM with enhanced regeneration.	[92]
Brazilian propolis	Hydrogel	Pullulan/PVA	Porous structure; sustained release; strong antioxidant activity; downregulated TNF-α, IL-6, IL-1β; antibacterial against *S. aureus* and *E. coli*; promoted collagen deposition and angiogenesis in diabetic rats.	Multifunctional hydrogel for diabetic wound healing with anti-inflammatory and pro-angiogenic activity.	[93]
Royal jelly (MRJP)	Photo-crosslinkable hydrogel	Major royal jelly proteins (MRJP)	Gelation within 2 s; hemostasis achieved in 43% of the time required for gelatin sponge; <5% bacterial survival on day 3; promoted CD206^+^ macrophage polarization.	Multifunctional hydrogel providing rapid hemostasis, infection control, and tissue repair.	[94]
Royal jelly	Hydrogel	Polyvinylpyrrolidone	Royal jelly increased swelling capacity and elongation while reducing tensile strength; homogeneous surface morphology and suitable degradation profile.	Flexible hydrogel dressing with improved physicochemical properties for wound care.	[95]
Royal jelly	Hydrogel film	Alginate/Gelatin	Young’s modulus 5–12 MPa; improved swelling and moisture regulation; enhanced fibroblast proliferation and migration; complete wound closure within 14 days.	Bioactive hydrogel film with antibacterial activity and accelerated chronic wound healing.	[96]
Beeswax + Naringenin/Ferulic acid	Dual-responsive hydrogel	Carboxymethyl chitosan/Poloxamer 407	Controlled release for 48 h; 92.4 ± 4.9% scratch wound closure after 48 h; 93.2 ± 3.4% wound closure on day 15; increased VEGF and reduced ICAM-1 (*p* < 0.001).	Smart pH- and temperature-responsive hydrogel for diabetic foot ulcers.	[97]
Bee venom	Hydrogel	PVA/Chitosan	Optimal formulation: 10% PVA + 0.6% chitosan + 4% bee venom; enhanced wound healing; anti-inflammatory effect comparable to diclofenac gel; increased hydroxyproline and glutathione; reduced IL-6.	Bee venom-loaded hydrogel with excellent mechanical properties and enhanced diabetic wound healing.	[98]

**Table 3:** Registered clinical trials evaluating beehive product-based interventions. Clinical trial information was obtained from the ClinicalTrials.gov registry.

Study/NCT Number	Study Design	Phase	Status	Enrollment (n)	Intervention	Wound Indication	Primary Endpoint/Key Findings
NCT06220344	Randomised, triple-blind, controlled trial	Phase II/III	Completed	80	Honey dressing vs. iodine dressing	Clean post-surgical wounds	Evaluated prevention of surgical-site infection, wound dehiscence and healing. Trial completed; results not yet posted.
NCT02259491	Randomised, single-blind controlled trial	Phase IV	Terminated	10	Medihoney^®^ dressing vs. standard wound care	Split-thickness skin graft and free flap donor sites	Assessed scar quality and postoperative pain; study terminated before completion.
NCT06150326	Randomised, single-blind pilot trial	Not Applicable	Recruiting	60	Activon^®^ medical honey vs. standard care	Acute wounds in ICU patients	Primary outcome is percentage wound surface reduction at Day 15; 3-month healing follow-up planned.
NCT01748318	Randomised, open-label controlled trial	Phase II	Recruiting	60	15 mL topical honey vs. oral Bactrim DS	Community-acquired MRSA abscess wounds	Evaluates lesion size reduction after 7 days compared with antibiotic therapy.
NCT03934281	Randomised controlled trial	Not Applicable	Completed	24	Thyme honey + hyaluronic acid dressing vs. standard dressing	Toe amputation wounds in diabetic patients	Primary endpoint: epidermization rate at 6 months following diabetic toe amputation. Results pending.
NCT04277182	Randomised controlled study	Not Applicable	Completed	36	Topical 10% and 15% propolis vs. silver sulfadiazine, nitrofurazone and controls	Second-degree superficial burn wounds	Histological evaluation of inflammation, angiogenesis, collagen deposition, epithelialization and oxidative stress biomarkers.

## Data Availability

No new data were created or analyzed in this study. Data sharing is not applicable to this article.

## Abbreviations

The following abbreviations are used in this manuscript:

ABTS	2,2′-Azino-bis(3-ethylbenzothiazoline-6-sulfonic acid)
AGEs	Advanced glycation end products
Ang-1	Angiopoietin-1
AAPH	2,2′-Azobis(2-amidinopropane) dihydrochloride
ATF-3	Activating transcription factor-3
BAX	BCL2-associated X protein
BCL-2	B-cell lymphoma 2
BD-2	β-Defensin-2
CAPE	Caffeic acid phenethyl ester
CCL2	C-C motif chemokine ligand 2
CXCL2	C-X-C motif chemokine ligand 2
DFU	Diabetic foot ulcer
EPE	Ethanolic propolis extract
EPCs	Endothelial progenitor cells
eNOS	Endothelial nitric oxide synthase
EVs	Extracellular vesicles
FRAP	Ferric reducing antioxidant power
GPx	Glutathione peroxidase
GR	Glutathione reductase
GST	Glutathione S-transferase
GAE	Gallic acid equivalents
ICAM-1	Intercellular adhesion molecule-1
iNOS	Inducible nitric oxide synthase
MAPK	Mitogen-activated protein kinase
MBC	Minimum bactericidal concentration
MGH	Medical-grade honey
MIC	Minimum inhibitory concentration
MMPs	Matrix metalloproteinases
NF-κB	Nuclear factor kappa B
Nrf2	Nuclear factor erythroid 2-related factor 2
SOD	Superoxide dismutase
Tie-2	Tyrosine kinase with immunoglobulin-like and EGF-like domains 2
TGF-β	Transforming growth factor-beta
TPA	12-O-Tetradecanoylphorbol-13-acetate
WVTR	Water vapour transmission rate
